# A situational analysis of training for behaviour change counselling for primary care providers, South Africa

**DOI:** 10.4102/phcfm.v7i1.731

**Published:** 2015-03-18

**Authors:** Zelra Malan, Bob Mash, Katherine Everett-Murphy

**Affiliations:** 1 Family Medicine and Primary Care, Stellenbosch University, South Africa; 2 Chronic Diseases Initiative in Africa (CDIA), Faculty of Health Sciences, University of Cape Town, South Africa

## Abstract

**Background:**

Non-communicable diseases and associated risk factors (smoking, alcohol abuse, physical inactivity and unhealthy diet) are a major contributor to primary care morbidity and the burden of disease. The need for healthcare-provider training in evidence-based lifestyle interventions has been acknowledged by the National Department of Health. However, local studies suggest that counselling on lifestyle modification from healthcare providers is inadequate and this may, in part, be attributable to a lack of training.

**Aim:**

This study aimed to assess the current training courses for primary healthcare providers in the Western Cape.

**Setting:**

Stellenbosch University and University of Cape Town.

**Methods:**

Qualitative interviews were conducted with six key informants (trainers of primary care nurses and registrars in family medicine) and two focus groups (nine nurses and eight doctors) from both Stellenbosch University and the University of Cape Town.

**Results:**

Trainers lack confidence in the effectiveness of behaviour change counselling and in current approaches to training. Current training is limited by time constraints and is not integrated throughout the curriculum – there is a focus on theory rather than modelling and practice, as well as a lack of both formative and summative assessment. Implementation of training is limited by a lack of patient education materials, poor continuity of care and record keeping, conflicting lifestyle messages and an unsupportive organisational culture.

**Conclusion:**

Revising the approach to current training is necessary in order to improve primary care providers’ behaviour change counselling skills. Primary care facilities need to create a more conducive environment that is supportive of behaviour change counselling.

## Abstrait

**Analyse situationnelle de la formation à donner aux fournisseurs de soins primaires en Afrique du Sud pour leur permettre de faire changer les comportements**.

**Contexte:**

Les maladies non contagieuses et les facteurs de risques associés (la cigarette, l'abus d'alcool, l'inactivité physique et un régime malsain) contribuent fortement à la morbidité des soins primaires et au fardeau de la maladie. Le Département de la Santé national reconnait le besoin de former des prestataires de soins pour modifier les modes de vie. Cependant, les études locales suggèrent que les conseils donnés par les prestataires pour changer les modes de vie sont insuffisants ce qui peut être attribué en partie à un manque de formation.

**Objectif:**

Cette étude avait pour but d’évaluer les formations actuelles pour les prestataires de soins primaires dans la province du Western Cape.

**Cadre:**

Université de Stellenbosch et Université de Cape Town.

**Méthodes:**

Des entrevues qualitatives ont eu lieu avec six informateurs importants (formateurs d'infirmières en soins primaires et secrétaires généraux de médecine familiale) et deux groupes de consultation (neuf infirmières et huit médecins) des Universités de Stellenbosch et de Cape Town.

**Résultats:**

Les formateurs manquaient de confiance dans l'efficacité des conseils à donner pour effectuer un changement de comportement et dans l'approche actuelle à la formation. La formation actuelle est limitée par des contraintes de temps et n'est pas intégrée dans tout le programme - l'accent est plutôt mis sur la théorie que sur le modèle et la pratique, et il y a un manque d’évaluation formatrice et globale. La mise en pratique de la formation est limitée par le manque de matériel éducatif pour les patients, la continuité des soins et la mauvaise tenue des dossiers, des conseils de mode de vie contradictoires et une absence de culture d'organisation.

**Conclusion:**

Il faut réviser la démarche actuelle de formation pour améliorer les compétences des prestataires de soins primaires qui aideront à faire changer les comportements. Les établissements de soins primaires doivent créer un environnement plus adapté qui soutient les services de conseils pour changer les comportements.

## Introduction

The burden of non-communicable diseases (NCD) is predicted to increase worldwide due to the ageing of populations, urbanisation and the globalisation of underlying risk factors. The rising morbidity and mortality related to NCDs has major implications for the delivery of acute and chronic healthcare services.^1^

The risk factors associated with NCDs have been clearly identified through international research and have been confirmed locally.^2,3^ Smoking, excessive alcohol consumption, physical inactivity and unhealthy diet are the key modifiable factors contributing to morbidity and mortality from NCDs.^4^ In South Africa, the burden of NCDs disproportionately affects the socio-economically disadvantaged and places increased demands on the public sector primary care services.^3^ Improving risky lifestyle behaviours is an important approach to decreasing health disparities and for more cost-effective utilisation of scarce resources in the public health sector.

Healthcare providers can play an important role in counselling and supporting patients with lifestyle risk factors or an established NCD.^3^ Patients have frequent contact with healthcare professionals, who are well positioned to provide counselling and who are also viewed by patients as being reliable sources of information. The best interface for this counselling in South Africa would be within public-sector, primary healthcare (PHC) services, as this is where the majority of the population encounters the healthcare system on a regular basis.^5^

Health services in low-/middle-income countries, such as South Africa, are based on a model of treating acute episodic illness and are not well organised for the prevention and management of NCDs. Counselling and education about risky lifestyle factors is usually inadequate.^1^ Until recently, the prevention of these lifestyle risk factors received little attention in South Africa's health-related priorities.^6^

Recently, however, the need for healthcare-provider training in evidence-based lifestyle interventions, both at an undergraduate level and as part of continuing professional development, has been acknowledged by the National Department of Health in their strategic plan for NCDs.^7^ In line with World Health Organization recommendations, the strategic plan prioritises cost-effective and feasible interventions to address the NCD epidemic.^8^ Brief behaviour change counselling in primary care is recommended for all four risk factors.^7,8^ Training healthcare providers in effective communication skills is seen as necessary and is particularly important, given the potential for prevention and control of NCDs at primary care level.^8^ A patient-centred approach, which actively engages the patient in decision making about their health, is seen as an objective in ‘re-orienting’ the PHC system to effectively address NCDs.^7,8^

To date, there has been very little research assessing the capacity of healthcare providers in South Africa to deliver behaviour change counselling, however these few studies suggest that counselling is inadequate.^5,9^ Although it is primarily nurses who provide behaviour change counselling in the public sector, nurses were found to have limited knowledge of how to counsel patients on NCD risk factors.^5^ Private general practitioners have also been found to struggle with documenting and counselling overweight or obese patients.^9^ Two other local studies, which investigated the knowledge, attitudes and practices of obstetricians and midwives respectively, around the delivery of smoking-cessation counselling to pregnant smokers, showed very low levels of counselling practice and knowledge of best-practice methods amongst both categories of healthcare providers.^10,11^ This poor performance amongst practitioners may reflect a lack of training in behaviour change counselling skills.

Brief behaviour change counselling is the ability to skilfully help patients change risky lifestyle behaviours. This study forms the first part of a larger research project, which aims to develop and implement a training intervention for PHC providers on brief behaviour change counselling, as well as to assess the provider's competency in delivering this counselling intervention. The aim of this study was to analyse the factors which were important for the design of a behaviour change counselling training programme in our setting.

The ADDIE (Analysis, Design, Development, Implementation and Evaluation) model provides a systematic approach to the creation of new educational programmes.^12^ This study reports on the analysis step of the ADDIE model, which helped the researchers to understand the current situation with regard to behaviour change counselling in our primary care context and the learning needs of primary care providers. The aim of this study, therefore, was to conduct a situational analysis of the current training courses and practice of primary care providers in the Western Cape with regard to brief behaviour change counselling. The specific objectives were:

- To explore the perceived effectiveness of current counselling and the factors that influence this.
- To explore the perceived effectiveness of current and prior training courses and the factors that influenced this.

## Research methods and design

### Study design

This was a qualitative situational analysis that made use of both individual in-depth and focus group interviews to explore the perceptions of primary care nurses and doctors as well as training programme coordinators. This situational analysis subsequently informed the design, development and evaluation of a new approach to training (using the ADDIE model), which will be reported on in future articles.^12^

### Setting

In the Western Cape Province, doctors are trained at Stellenbosch University and the University of Cape Town. After undergraduate studies, internship and community service, doctors can choose further postgraduate training. Postgraduate training for primary care is via a four-year Masters of Medicine degree in family medicine. Basic training for nurses is offered by universities (such as the University of Western Cape) and nursing colleges. After basic training, nurses can qualify as a clinical nurse practitioner through a one-year Higher Diploma course, which is offered by Stellenbosch University and the Western Cape College of Nursing.

Primary care services are offered via a network of fixed and mobile clinics as well as community health centres throughout all the health districts in the Province. Patients with NCDs are mostly managed in these facilities. At clinics, the service is offered by a nurse with periodic support from a doctor. At health centres, service is also offered mainly by nurses, but with more involvement from doctors as well as a broader multidisciplinary team that might include health promoters, occupational therapists, physiotherapists and pharmacists.

In qualitative research, the degree of objectivity of the researcher can partly be judged by their own self-awareness and relationship to the topic. The researcher in this study is a qualified family physician and spent many years working in private general practice. She developed an interest in behaviour change counselling through previous research on general practitioners counselling overweight and obese patients,^9^ which prompted her to think about possible training interventions that could improve the counselling skills of PHC providers.^9^

### Study population and selection of participants

Key informants were selected on the basis that they coordinated postgraduate training for either primary care nurses or registrars in family medicine in the Western Cape. Interviews were conducted with the two programme managers involved in the training of nurses for the Diploma in Clinical Nursing Science, Health Assessment, Treatment and Care at Stellenbosch University (this course is not offered at the University of Cape Town) as well as three programme managers involved with the training of registrars in Family Medicine at Stellenbosch and Cape Town Universities. An additional family physician working at a public PHC facility in the Western Cape, who supervises registrars and is actively involved in research on teaching and assessment of registrars, was interviewed to gain a complete picture of current training in behaviour change counselling in the clinical training environment.

Focus group interviews were conducted with nurses and doctors currently working in primary care and who had experience of these training programmes. Nine nurses working at a primary care clinic, situated in a low socioeconomic area of the Cape Winelands, were selected. The researcher was familiar with the nursing staff at this clinic, as she has been involved with the training of undergraduate medical students at this facility on a weekly basis, for the last few years. The nurses were established primary care providers and were trained in the Western Cape. They were familiar with the community and had been working at their facility for more than a year. All nurses were interviewed to get an overall view from each level of trained nurse. Four of the nurses were clinical nurse practitioners, four were staff nurses and one was an assistant nurse.

The second focus group interview was with a group of eight registrars in family medicine at the University of Cape Town. The registrars ranged from first- to third-year students, had previously been working as junior doctors and had received their undergraduate training at a variety of different universities. At the time of the research it was logistically difficult to conduct a focus group interview with Stellenbosch registrars.

The design allowed for further interviews to be conducted if the analysis and triangulation of these initial interviews suggested the need to identify and explore additional issues.

### Data collection

The researcher performed and audio-taped in-depth interviews with all the key informants listed above. Interviews were conducted in the key informant's choice of language, either Afrikaans or English. The researcher used an interview guide and skills such as open-ended questions, reflective listening, summarising and elaboration to conduct the interview. Topics discussed were: the current teaching modules, if any, on behaviour change counselling; prior experiences and beliefs about the effectiveness of such training; attitude toward the introduction of a new training module; and their beliefs about the long-term effect of current training in clinical practice.

The focus group interviews were also conducted by the researcher and audio-taped. The interview with the nurses took place at the clinic and the interview with the registrars at the university campus. The nurses preferred to be interviewed in Afrikaans and the registrars in English. During these interviews the researcher used an interview guide to explore their successes and failures with counselling, their perceptions of factors that enabled or obstructed counselling and their perceived effectiveness/competency in delivering counselling. Participants were also asked about prior formal training received and perception of their knowledge on NCDs.

### Data analysis

All interviews were transcribed verbatim and analysed using Atlas.ti software (v. 6.2.15 2011) using the framework approach.^13^ The framework approach to content analysis involves the following steps:

- Familiarisation: The researcher listened to the tapes, read the transcripts and listed recurrent issues or ideas that emerged from the data.
- Construction of thematic framework: The researcher organised these issues and ideas into a framework that was aligned with the interview guide and objectives of the study. In Atlas.ti, this related to a list of codes organised into families.
- Coding: The researcher applied the thematic framework systematically to all the data by annotating the transcripts with the codes using Atlas.ti.
- Charting: All the data from the specific codes included in a family in Atlas-ti were brought together in one document or chart.
- Mapping and interpretation: The researcher used the charts to interpret the data for themes and look for any associations or relationships between themes.

All three researchers were involved in this process: familiarisation, coding, construction of the thematic framework, charting and interpretation of the charts.

### Ethical considerations

This study was approved by the Health Research Ethics Committees (HREC) at Stellenbosch University (Reference number: N11/11/321). Key informants and focus group participants gave written consent. The confidentiality and privacy of all interviewees and participants were respected in data analysis and reporting.

## Results

Overall, 23 people were interviewed, comprising six key informants, nine nurses working in primary care and eight doctors training in family medicine and primary care, as described in the methods. Fifteen of the respondents were women and eight were men, with ages ranging from 24 to 56 years.

Both doctors and nurses believed that healthcare providers (HCPs) should be skilful in their ability to help patients make difficult decisions about changing risky lifestyle behaviours:

‘Everybody needs to be able to do it and do it effectively.’ (Nurse programme manager)

‘I think it is a crucial part of the skills set that any family physician should have.’ (Family medicine programme manager)

‘We can't just waste it, in the sense of giving more medication, but the cause of the problem is not addressed [*through counselling*].’ (Nurse)

Although it was seen as an integral and important part of an HCP's competencies, nobody expressed confidence in the current training or its impact on practice in the clinical setting:

‘So we haven't found a form of behaviour change counselling that really works and can work at scale in the context of our primary care scenario, where things are very pressurised, you know, and with nurses it is a huge challenge.’ (Family medicine programme manager)

‘The current training programmes do not meet the needs of the country.’ (Nurse programme manager)

‘We have not gone as far as changing the clinical picture.’ (Family physician)

The current training of registrars was seen as being mainly theoretical and did not enable the development of practical skills. Time constraints, as a result of other competing issues in the curriculum, resulted in a lack of continuity throughout the curriculum and made it difficult for HCPs to fully integrate new skills into clinical practice. Registrars felt that the limited time spent on training in behaviour change counselling led to the impression that it is of lesser importance:

‘So I am very concerned about how little time they actually have and you know, what they are picking up and then even more concerned about whether they will practise in any of that skills, in a way that will encourage you know their on-going learning.’(Family medicine programme manager)

‘We just had one session which makes it feel almost as if it's of lesser importance. It's *ja*, so and we're always told you know, you need to counsel your patients its important and I don't think we get taught enough about it undergrad and postgrad.’ (Registrar).

Nurses on the one-year diploma course had only seven contact sessions of two hours allocated to the module, Principles and Processes of Primary Care; and the training in this module focused on breaking bad news, substance abuse and intimate partner violence. During this module there was virtually no training or assessment of behaviour change counselling skills. They did role play and audio-taped a normal consultation with a patient, then received feedback from the lecturer on general communication skills. Final assessment of their communication skills involved a single role play that did not necessarily focus on counselling. Nurses were thought to be starting at a much lower level in terms of their prior communication skills because less focus was placed on these skills in their basic training and they had less experience of consulting in their work environment prior to training as primary care nurses. Typically, the nursing consultation tended to be more task oriented, which made it difficult for them to adopt a holistic patient-centred approach. During their training they were not taught knowledge related to risky lifestyle behaviours for NCDs, but rather a general approach to communication. It was apparent that lecturers did not expect nurses to be competent after the training and also that there was no follow up after completion of the training programme:

‘What tends to happen is that because it is so short and because some of them start with a very low baseline, it is difficult to get them to significance.’ (Nurse programme manager)

‘Remember our students have only seven contact sessions in which we need to teach them everything. You must understand that everybody wants their specific thing to be concentrated on and we only have seven lectures.’ (Nurse programme manager)

‘I don't teach them to become professional counsellors at all, it's teaching for concepts.’ (Nurse programme manager)

Family medicine lecturers commented on an organisational culture in the health services whose values were often incongruent with the style required for behaviour change counselling, namely, one that is respectful, empathic and collaborative. Lack of support from clinic managers for behaviour change counselling and modelling of an authoritarian and directive style of communication, made it challenging for HCPs to implement any training they received:

‘How do you get a health worker to behave in a guiding style, in an organisation that manifests values almost directly opposite to those values you know?’ (Family medicine programme manager)

Registrars at both universities were exposed to communication skills training, including motivational interviewing, early in their four-year programme. The training was over a three-month period in first year, as part of a consultation module. This actually involved a 1–2 week section where they studied basic motivational interviewing (MI). This involved several readings, watching a video that modelled the counselling approach and a written assignment that reflected on attempts to counsel behaviour change at the end of the module. During the second year, registrars at Stellenbosch attended a one-day workshop on brief MI. The registrars at Cape Town developed and practised consultation skills by using audio-taped role plays with no specific focus on counselling. After the more formal teaching in the first and second years there was no specific requirement for these counselling skills to be observed or reinforced as part of their work-based training and portfolio assessment, although the portfolio does require observation and feedback on consultation skills in general.

Registrars at both universities were assessed in fourth year on their ability to perform behaviour change counselling in a simulated consultation, which is one out of four such consultations in the final examination, all of which focus on communication skills. In the new national exit exam for family medicine, organised by the College of Family Physicians, candidates are required to perform three observed consultations and the assessment tool includes an assessment of their general counselling skills. This section of the tool, however, defines counselling more in terms of general health education than behaviour change counselling:

‘So what they really have taken out, and what is actually incorporated into their consultation, is not known.’ (Family medicine programme manager)

‘We have not gotten [*sic*] as far as saying we want to be absolutely sure that every registrar is competent in that skill.’ (Family medicine programme manager)

‘It's really difficult to assess.’ (Family physician)

Although feedback is considered an important factor in developing and maintaining a new skill, once registrars and nurses were in actual clinical training practice, no feedback on their skills was available, mainly because most supervisors had not received training in specific counselling skills:

‘How confident are you at using it and if you don't get feedback on your success at using it, it is very difficult to develop enough confidence to carry on trying to use that skill.’ (Family medicine programme manager)

‘They're from all over the country and so we don't know, they pass the programme, they get their diploma and they're out of here.’ (Nurse programme manager)

‘It is one thing to be taught how to do it, it is another thing actually changing your practice to actually doing that. It is much easier probably for people to just go back to doing things the way they have always done them.’ (Family medicine programme manager)

‘In terms of the registrar's supervision, absolutely no idea if any of the supervisors do any teaching around brief motivational interviewing at the training sites.’ (Family medicine programme manager)

Doctors expressed the need for feedback in future training:

‘I think it will be a big help if we have to counsel someone with a supervisor or somebody or a lecturer watching us and giving feedback.’ (Registrar)

‘When you are trying to use a new technique which you have admittedly only had six hours training in, how confident are you at using it and if you don't get feedback on your success at using it, it is very difficult to develop enough confidence to carry on trying to use that new skill.’ (Family medicine programme manager)

As a result, doctors and nurses lacked confidence in their counselling skills and did not feel equipped to counsel effectively:

‘So it is not an easy procedure to verbally tell someone you must stop smoking.’ (Nurse)

‘I cannot just tell a person to quit smoking; there is no way that you can just quit smoking.’ (Nurse)

‘The only counselling skills that I know is like HIV counselling skills or the counselling of the dying patient, but not really the steps of making someone stop smoking.’ (Registrar)

‘We have been taught but you don't actually, I don't think I'm confident enough to do it in the right way, I'm doing it in the way that I feel is best.’ (Registrar)

Nurses did not recall being trained to counsel a patient to change risky behaviour, thus basing their current counselling methods on their own past experiences. Counselling was viewed as part of the consultation and not a specific technique. Nurses used a more directive style, where asking and informing patients about the risk factor seem to be the dominant skills used. There was a perception that one had to use a direct style if you had limited time. Most of the knowledge used during this counselling was reportedly obtained from magazines, the radio and newspapers:

‘We have taught ourselves with experience.’ (Nurse)

‘It's part of how we anyway see a patient.’ (Nurse programme manager)

‘There is no specific technique, actually we just talk.’ (Nurse)

‘I ask are you smoking, are you drinking, then I try to convince them that it is not good.’ (Nurse)

‘I ask them a question and I inform them what the risks are towards that. For example, I ask “do you smoke?”’ (Nurse)

‘To do it all in five minutes you have to be direct.’ (Nurse)

Registrars tried to involve the patient in decision making, but still relied on information giving when counselling, using a brief 1–2 minute intervention as part of the consultation. Interestingly, both nurses and doctors felt more comfortable with regard to counselling a patient on diet and exercise than with regard to advising them to stop or reduce smoking tobacco or drinking alcohol. There was a perception that you would be wasting your time trying to counsel someone on tobacco smoking or alcohol use:

‘I make them aware of what I think needs attention and we have learnt with, like, getting the patient to participate in the decision making and sometimes I will ask, “so what do you think you can do differently?”’ (Registrar)

‘So it's easier to tell someone to eat salad, he will more willingly eat salads and tomatoes and stuff like that, than you telling him to quit smoking.’ (Nurse)

‘I feel sometimes diet, people can change and exercise, but smoking sometimes I just feel like someone's going to smoke anyway.’ (Registrar)

Nurses felt overwhelmed in a situation where they were expected to counsel, lacked practical skills, had time constraints and pressure of workload, lacked appropriate support materials and felt that patients were likely to be irresponsible anyway, despite knowing the risks:

‘They have the information but they are still smoking. They see it on television, they see it on the cigarette packet, they see it in the newspaper, in a book they are reading and we tell them. They still smoke.’ (Nurse)

‘It will be much easier with what you say to have colourful pictures, because there are people who can't read.’ (Nurse)

‘So you need to give them something to read about the danger of smoking and all that stuff, it would be much better.’ (Nurse)

‘How can we do all of this in five minutes?’ (Nurse)

Despite these barriers, registrars remained sympathetic toward patients’ circumstances, but also felt that poverty made it difficult to adopt a healthier lifestyle:

‘A lot of times our patients’ lives are just so miserable, like they are just so poor and so like the areas they live are such bad areas and, for example, if someone smokes then sometimes I feel like *ag* shame just let them smoke, like, it's their only little pleasure.’ (Registrar)

‘You can't tell a diabetic that doesn't have an income and three children with only child support grants they must have a low GI [*glycaemic index*] diet. I mean they eat what they can to stay alive.’ (Registrar)

Doctors reported poor continuity of care, poor record keeping, lack of a standardised approach, language and cultural issues as being additional difficulties. Both doctors and nurses expressed difficulties in counselling a patient when they themselves were smokers or overweight. Doctors felt that nurses had a better understanding of the patient's language and culture because they often stay in the community, but sometimes lacked the knowledge on risk factors and NCDs when counselling. This also led to the problem of HCPs giving conflicting or contradictory messages:

‘Continuity of care, we never see the patient twice so you can't really say let's talk a little bit today and then next week we will continue …’ (Registrar)

‘You don't always see the notes, or recordings about previous brief things are not always in the notes.’ (Registrar)

‘They understand a person's culture better and when you work with them although sometimes a problem is also that sometimes their knowledge might not be adequate enough and then we get a problem where they say one thing, you say another.’ (Registrar)

Doctors did not think counselling alone would be sufficient to change a patient's behaviour and that it needed to be combined with giving printed patient information material. They also felt that patients preferred to see a doctor and trusted their advice more than the nurses, although it was not clear if this was because of the counselling style, higher professional status of doctors in the patient's perspective or for some other reason:

‘If it comes from the doctor's mouth then it's like, no, this is definitely the correct thing I'm not listening to the nurse. Unless they've actually bonded with the nurse or they have had a good experience or with someone that's very patient-centred, but from my experience patients tend to prefer doctors.’ (Registrar)

‘I think they remember more if you actually tell them rather than say here's the pamphlet, go read up on hypertension and diet or something.’ (Registrar)

Nurses felt that they should target young people in future because old people are set in their ways and less likely to change. They reported that counselling for smoking-cessation in future could be more successful if the patients are prescribed medication to assist them in changing their behaviour, but unfortunately not many could afford it:

‘Our major problem is that we don't start with the right people. We must start with the children.’ (Nurse)

‘So you must start from where you started smoking, people from 20 or between 25 or wherever. If you are 14 you smoke because it is fun, your friends are smoking, but if you are smoking at the age of 20, you smoke because you smoke.’ (Nurse)

‘Patients that really want to stop ask the doctor to write a prescription that they get at the private chemists and it is a bit expensive but those that are given prescriptions, were successful and they wanted to stop.’ (Nurse)

The nurse programme managers felt that counselling skills need to be integrated into current nursing practice in order to make the time available for counselling more skilful and effective. Trying to implement counselling as an additional task would not be successful:

‘You have to incorporate whatever you are doing into what the nurses are already doing, because you're not going to get it done separately. They don't have the time or the interest, and how to, and you know every time you add something to a nurse's workload they tell you, okay so where am I going to fit this into my day?’ (Nurse programme manager)

A simple unified structured approach based on the best evidence available should be adapted and implemented in the primary care system. Future training should be aimed at improving supervisors and other HCPs counselling skills at undergraduate and postgraduate levels. Competence after training should be assessed and the importance of brief behaviour change counselling (BBCC) needed to be stressed by both policy makers and managers at the clinic level:

‘We must seriously look at integrating some of these things that only happen at the end and at the very beginning, integrate that far more into a form of a workplace based assessment throughout the four years.’ (Family medicine programme manager)

‘We need much more regular training and much more regular assessment.’ (Family medicine programme manager)

‘So having a simple structure that people can remember and apply almost generically to behaviour change issues that, could actually be highly beneficial in getting people to do this.’ (Family physician)

## Discussion

**FIGURE 1:**
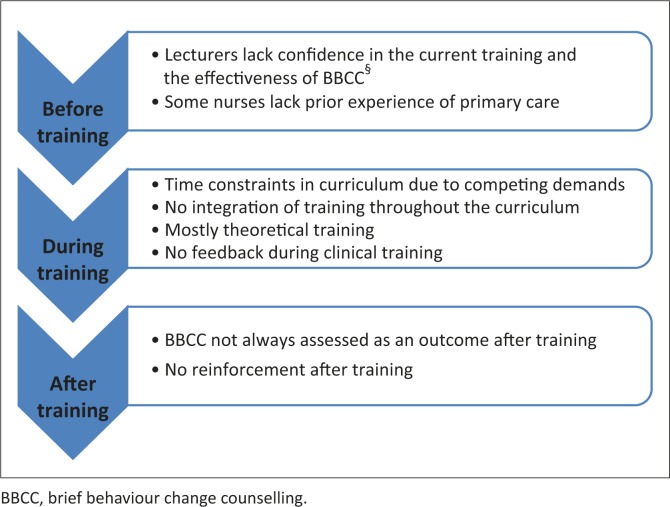
Current training difficulties.

Training of primary care nurses and family physicians in BBCC is not designed to really build competency. Training programmes seem to be promoting the theory of lifestyle modification, but are not delivering on the practical skills. The opportunity to practise key skills and to receive constructive feedback on performance is largely missing, thus HCPs do not transfer these skills to clinical practice. As a result, HCPs lack confidence in their ability to counsel patients effectively. The current difficulties with training are summarised in Figure 1.

The Department of Health's recommendations to revitalise PHC focuses on improving PHC providers’ capacity to counsel effectively, by teaching HCPs to deliver personalised, patient-centred behaviour change counselling.^7^ Traditionally, HCPs rely mostly on the directive style when counselling patients on behaviour change, resulting in resistance from the patient and frustration for the HCP.^5,14,15^ A patient-centred approach to counselling patients on lifestyle change outperforms a directive, advice-giving approach in 80% of studies.^16^ Involving the patient in decision making is essential in order to create a collaborative, culturally relevant and efficient interaction, especially in our diverse context.

Brief behaviour change counselling is built on the foundation of a patient-centred style and incorporates a guiding style derived from MI.^17^ It is designed for use in PHC settings, with brief interactions in mind.^4,17^ This study echoed other research, which showed that many programme managers and HCPs are unaware of the evidence in support of BBCC and are sceptical about its effectiveness.^5,18,19,20^ International evidence exists to show that BBCC can be delivered by a range of healthcare providers with minimum investment of time, in a variety of settings, for patients of different ages, genders and ethnicities.^21,22^ Healthcare providers from different training backgrounds, working in different settings, devoting a small amount of extra time with their patients and building a patient-centred relationship, can expect 10% – 15% additional improvement in patients across a wide variety of behaviours.^21,22^ This evidence can possibly be used to increase HCPs’ awareness of their potential role and to build their confidence as well as to sensitise programme managers and decision makers to the potential value of BBCC.

The relevance and applicability of BBCC has not been widely assessed in low- and middle-income country settings. The first attempt to apply BBCC skills in a primary care setting in a developing country such as South Africa in 2004, demonstrated that it has great potential for general practitioners.^17^ They felt less frustrated with behaviour change consultations and reported having more skills in counselling for behaviour change.^17^ During 2008, 38 lay- and nurse counsellors were trained to counsel pregnant mothers about behaviour changes related to the prevention of mother to child transmission of HIV in sub-Saharan Africa.^23^ This research developed recommendations to guide the development of future training programmes in this setting. One of the key messages was to tailor-make the training according to the HCP's baseline communication skills. Other recommendations included avoiding reinforcing problems, deficiencies and failures of the counsellors, but rather focusing on successes and aiming to build self-confidence.^23^

**FIGURE 2:**
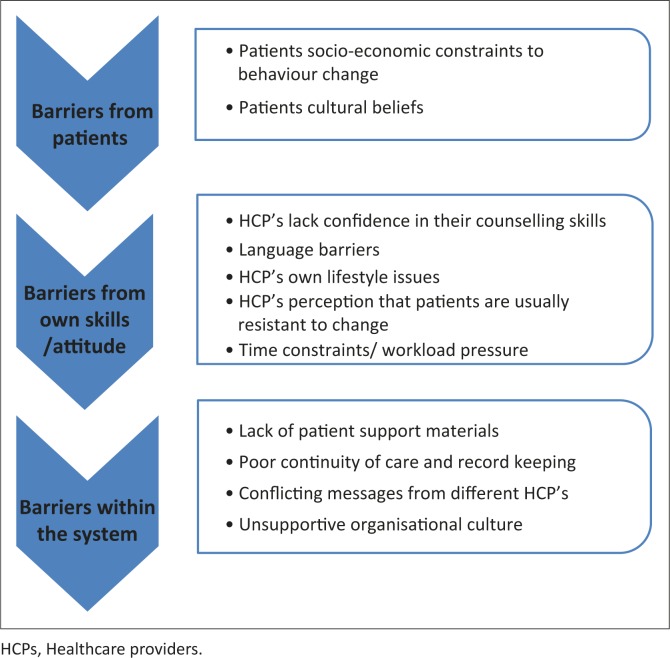
HCPs’^§^ barriers to behaviour change counselling in clinical practice.

Healthcare providers in practice reported a number of barriers to the delivery of BBCC, such as a lack of time, lack of confidence in their ability to counsel and a lack of supportive materials for patients (see Figure 2). There was a perception that counselling was ineffective, with poor patient adherence; and language barriers were also amongst the main difficulties in counselling. These same barriers have been reported in similar studies from the same setting.^5,20^

Even if current training programmes are adapted and optimised, we are still faced with numerous challenges in our public, PHC system that may be a barrier to HCPs’ ability to engage in patient-centred counselling. A recent study in the Western Cape reported that three-quarters of junior doctors working in community-level PHC facilities, are suffering from clinically-significant burnout.^24^ Emotional exhaustion and depersonalisation may diminish HCPs’ commitment to engage patients in patient-centred counselling.^24^

Future training could optimise training outcomes by targeting HCPs with the most potential to provide successful BBCC. Empathy has been identified as one of the essential skills valued most by patients, in communicating with a healthcare provider who uses a patient-centred approach.^25,26^ Healthcare providers who are low in empathy show indifference or active dismissal of the patient's perspective, whereas those high in empathy are curious and spend the time required to explore the patient's story. Patients experience this attitude as positive: they feel listened to, which then leads to an increased possibility of change.^26^ Recent international research suggested that training can make a significant difference in PHC providers’ empathic expression during patient interactions.^25^ Screening before training, as well as offering additional input as required, may be necessary in order to optimise gains in patient-centred communication skills training for counsellors with lower baseline empathy.^25^

The development of future training programmes should take note of current counselling barriers and aim to reduce those barriers that can be addressed through training skills. Training can teach communication skills and can change the HCP's attitude about the effectiveness of counselling.^15,25,27,28^ Doctors with good communication skills identify patients’ problems more accurately.^27^ In addition, they have greater job satisfaction and less work stress.^27^ The inclusion of training modules on communication skills for primary HCPs is therefore essential.^6,15,18^

Group education could also be explored as a possibility to deal with some of the barriers in PHC settings with limited resources. Health professionals regard group interactions as the most practical approach to counselling in our demanding primary care clinic setting. The first trial in an African context, on the effectiveness of a group diabetes education programme in the Western Cape, demonstrated that healthcare promoters have the potential to deliver effective group diabetes education.^16^ A combination of both structured and systematic group education, together with more ad hoc individual BBCC, could be a model to explore further.

Training has very limited impact on practice if there is no follow-up support and feedback.^23,29,30^ Offering feedback on real consultations could ensure more effective transfer of skills after initial training.^27^ Formative feedback is an essential part of a supervisor's role and including observation of BBCC in the registrar's portfolio of learning (the portfolio is now a national requirement in South Africa, for entry to the college exams) could strengthen the training in this area. The portfolio requires direct observation of consultations with feedback and could require that some of these focus more on BBCC. However, supervisors should also be trained on the best evidence-based methods for BBCC. In-service training and ongoing support can be effective in overcoming some of the barriers and improving clinician's provision of behaviour change counselling.^18,23^

### Limitations of the study

Interviews were conducted by the researcher, who is a family physician at the Division of Family Medicine and Primary Care at Stellenbosch University. Previous research conducted by the researcher on general practitioners’ management of overweight and/or obese patients, could have had a negative influence on her perception of the primary HCP's efficacy in counselling. The researcher has been involved primarily as the interviewer in all the interviews undertaken for this study, as well as the analysis and interpretation of the findings. The interview process, analysis and interpretation were, however, supervised by the other co-authors.

The researcher triangulated data from different types of respondents (trainers, nurses and doctors), from different genders, ages, institutions and with different levels of expertise and qualifications. However, a broader selection of study participants could have added to the credibility of the results. For example, focus group interviews with nurses from other clinics or with other primary care doctors outside of the training programme might have revealed additional themes.

### Recommendations

Based on the current training challenges identified by this study, future BBCC training programmes should aim to:

- Raise awareness of the evidence on the benefits of BBCC amongst primary care providers and decision makers.
- Include the evidence base for BBCC in the training.
- Focus on the development of competency in BBCC, rather than the theories of behaviour change, or general communication skills. Include time to both model and practise skills in the training.
- Reinforce the initial training provided throughout the rest of the programme.
- Ongoing, on-site feedback and supervision should be provided in the clinical training setting.
- Competency in BBCC should be assessed summatively as part of the programme.
- Based on the barriers to BBCC in clinical practice, the following recommendations can support the implementation in primary care:
  - Provide patient education materials to reinforce and supplement BBCC on risk factors and key behaviours.
  - Encourage record keeping and continuity of care to enable follow up of BBCC interventions.
  - Ensure that all relevant health workers are trained in BBCC and share the same understanding of lifestyle modification messages.
  - Develop an organisational culture that is patient-centred and which encourages learning and innovation so that BBCC is congruent with this culture.

Future research in our setting will focus on the development and implementation of a training intervention for primary HCPs on BBCC, based on the findings of this study and best-practice models.

## Conclusion

Current training on behaviour change counselling for primary care providers in the Western Cape is viewed as insufficient to achieve competence in clinical practice. Primary care providers’ current experience of counselling in practice tends to be discouraging and challenging, in view of the numerous barriers that they face. Revising the approach to current training is necessary in order to ensure that skills can be learnt and transferred to the clinical setting.
